# Programmable Cell-Free Transcriptional Switches for Antibody Detection

**DOI:** 10.1021/jacs.1c11706

**Published:** 2022-03-22

**Authors:** Aitor Patino Diaz, Sara Bracaglia, Simona Ranallo, Tania Patino, Alessandro Porchetta, Francesco Ricci

**Affiliations:** Department of Chemistry, University of Rome, Tor Vergata, Via della Ricerca Scientifica, Rome 00133, Italy

## Abstract

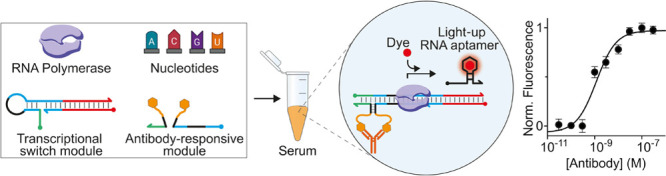

We report here the development of a cell-free *in vitro* transcription system for the detection of specific target antibodies. The approach is based on the use of programmable antigen-conjugated DNA-based conformational switches that, upon binding to a target antibody, can trigger the cell-free transcription of a light-up fluorescence-activating RNA aptamer. The system couples the unique programmability and responsiveness of DNA-based systems with the specificity and sensitivity offered by *in vitro* genetic circuitries and commercially available transcription kits. We demonstrate that cell-free transcriptional switches can efficiently measure antibody levels directly in blood serum. Thanks to the programmable nature of the sensing platform, the method can be adapted to different antibodies: we demonstrate here the sensitive, rapid, and cost-effective detection of three different antibodies and the possible use of this approach for the simultaneous detection of two antibodies in the same solution.

## Introduction

Diagnostic tests that are easy to perform, convenient, reliable, and suitable for use at the point of care are crucially needed in the detection, monitoring, and containment of infectious diseases and other clinical emergencies.^1−4^ In recent years, the possibility to couple the advantages of synthetic nucleic acids (i.e., programmability of interactions, low-cost, and ease of synthesis) together with the sensitivity offered by cell-free transcription/translation systems has led to the development of innovative sensors for the detection of different targets.^5−14^ Synthetic genetic circuits and switches that respond to specific DNA or RNA sequences,^5−8^ small molecules,^9,10^ and metal ions^11^ and trigger the cell-free transcription of signaling RNA aptamers or the translation of reporter proteins have led to analytical devices with excellent sensitivities and specificities. Recently, cell-free diagnostic tools for the detection of SARS-CoV-2 viral RNA fragments have been found useful even in the current COVID-19 pandemic.^13,14^

Despite the above advances, the examples reported so far of cell-free nucleic acid diagnostics have been demonstrated for a limited number of targets. The potentialities offered by responsive nucleic acid devices have thus not yet been fully exploited. Synthetic nucleic acid strands can be used as molecular scaffolds to append different recognition elements that allow the design of nucleic acid probes responsive to a wide range of targets.^15−19^ In a demonstration of such potentiality, we and others have recently employed antigen-conjugated nucleic acids rationally designed to respond to clinically relevant antibodies.^19−23^

Motivated by the above considerations, we demonstrate here a cell-free diagnostic platform for the detection of specific antibodies in blood serum based on the use of antibody-responsive nucleic acid transcriptional switches. The approach we propose couples the advantageous features of responsive DNA-based conformational switching probes with those of cell-free diagnostic methods in which target-induced transcription/translation of signaling RNA aptamers or proteins is used for detection purposes.

## Results and Discussion

### Sensing Principle of Antibody-Responsive Transcriptional Switches

**Figure 1:**
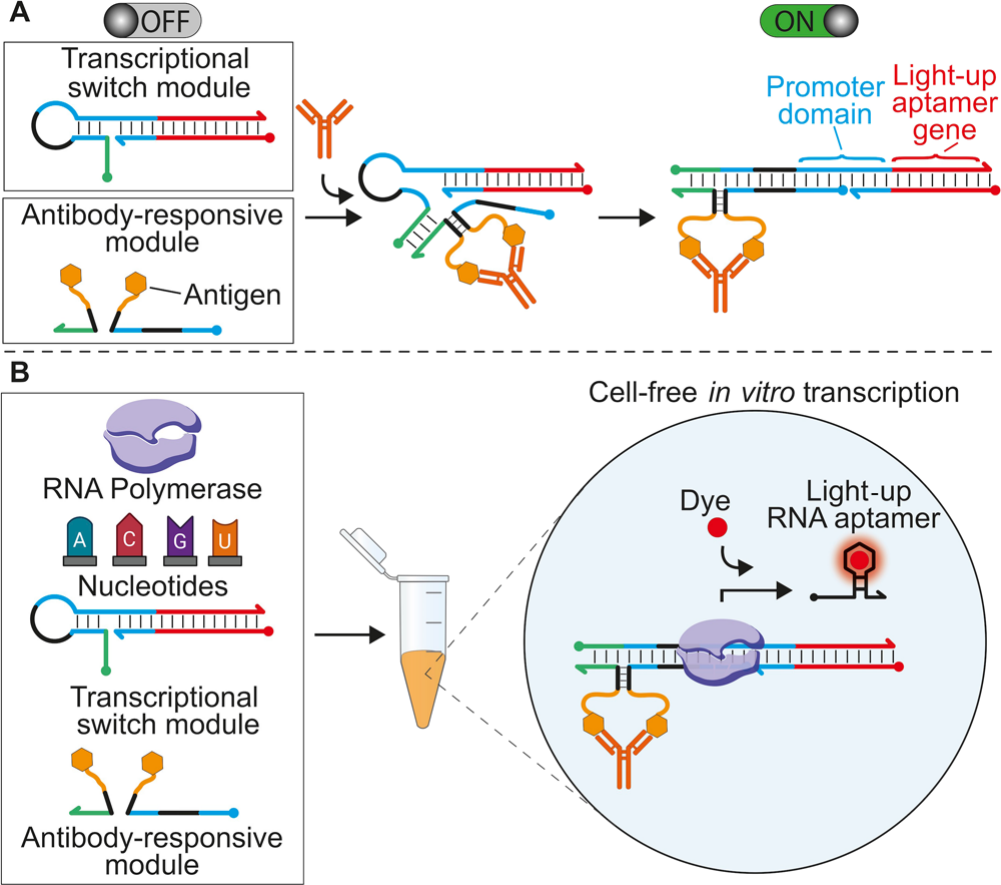
Programmable antibody-responsive transcriptional switches. (A) The antibody-responsive transcriptional switch is composed of two modules: the transcriptional switch module and the antibody-responsive module. The first is a double-stranded DNA switch designed to adopt a stem-loop hairpin conformation that prevents efficient transcription of an RNA light-up aptamer due to the incomplete formation of the promoter sequence. The second module is composed of two antigen-conjugated DNA strands (split input strands) that, upon bivalent binding to a target antibody, are brought into close proximity and can hybridize to form a functional bimolecular complex. Such a complex induces a conformational change on the switch and makes the promoter sequence accessible for transcription (right). (B) The so-activated transcriptional switch can transcribe, in the presence of RNA polymerase and nucleotides, a reporter light-up RNA aptamer that signals the presence of the target antibody.

Our strategy to achieve an antibody-responsive transcriptional switch is based on the use of a pair of DNA-based functional modules that are rationally designed to trigger the transcription of a signaling RNA aptamer in the presence of a specific target antibody (Figure 1A). The first module of this platform is the transcriptional switch, a conformational-switching hairpin DNA composed of two complementary DNA strands (Figure 1A). Such a transcriptional switch consists of three major domains: a double-stranded (ds) portion encoding for the RNA output of the switch (red domain), a T7 RNA polymerase (RNAP) promoter domain (blue), and a switching domain encoded in the stem-loop structure of the hairpin. The output of the transcriptional switch is a light-up RNA aptamer such as a Mango or Spinach aptamer.^24−26^ The switch is designed so that, in the absence of the specific target antibody, the transcription of the output RNA aptamer is inefficient due to the incomplete nature of the promoter domain.

The second module of the platform is the antibody-responsive module composed of two antigen-conjugated DNA strands (here also named split input strands). These strands are rationally designed so that the hybridization of their complementary portions (black, Figure 1A) leads to the formation of a bimolecular functional complex (input) that is able to induce a conformational change in the transcriptional switch through a toehold strand displacement reaction. Such a conformational change leads to the formation of a complete promoter domain that can be recognized by T7-RNAP and induce efficient transcription (Figure 1A, right). The complementary portions of the antigen-conjugated DNA strands, however, are designed to form an unstable complex under the diluted concentrations employed. Only upon bivalent binding of the target antibody to these two strands, a co-localization-induced formation of the functional complex (input) is achieved, ultimately triggering the reaction with the transcriptional switch (Figure 1B). The modules can be used with a commercially available cell-free transcription kit (containing T7-RNAP, the nucleotides, and other components), and the fluorescence signal generated in the presence of the aptamer-binding dye will inform on the presence and concentration of the target antibody (Figure 1B).

### Design of Transcriptional Switches

**Figure 2:**
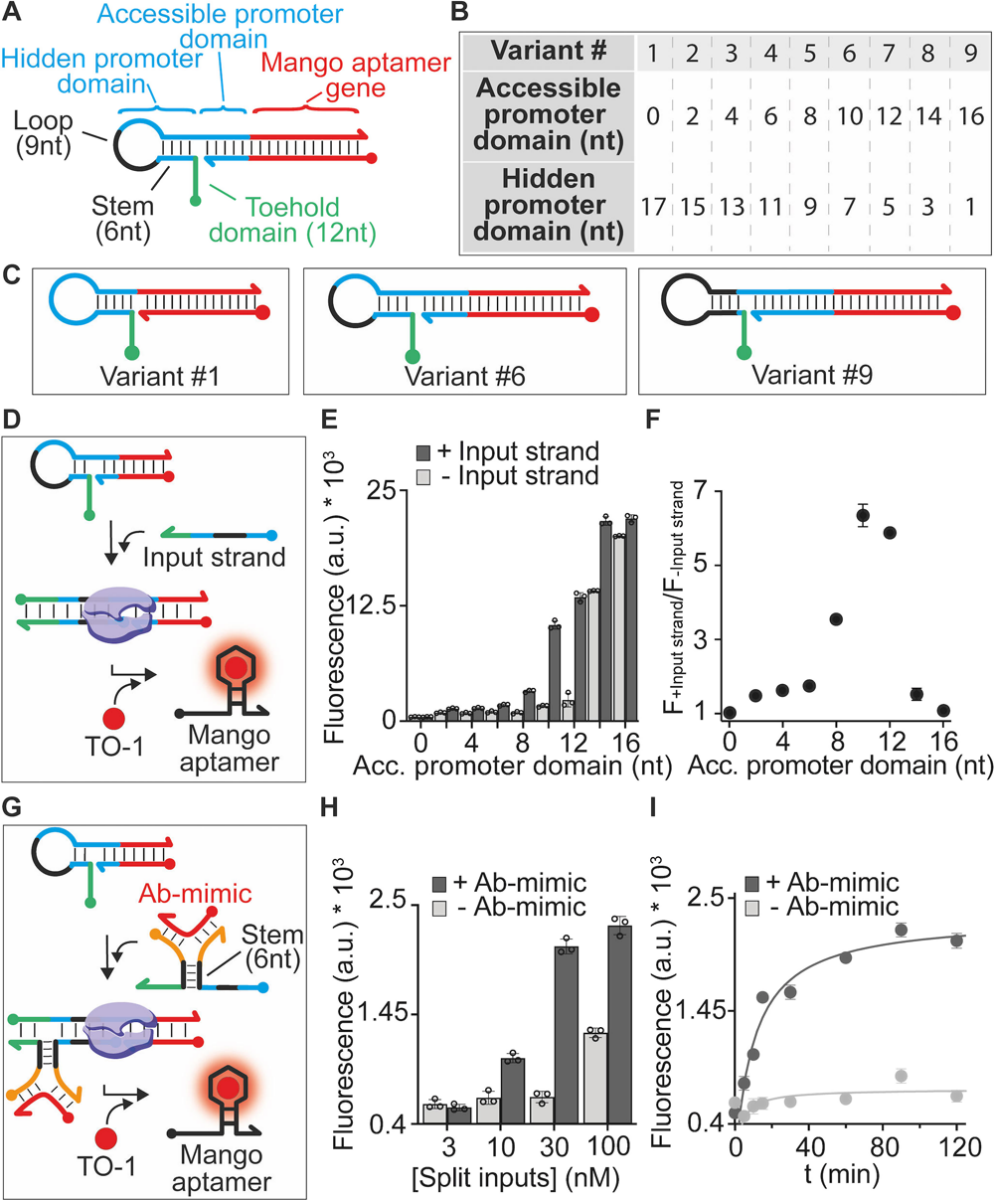
Design of the co-localization-induced transcriptional switch. (A) Scheme of the transcriptional switch with relevant functional domains indicated. (B) Table of the different variants tested and their corresponding length of the accessible and hidden promoter domain. (C) Three representative transcriptional switch variants (#1, #6, and #9). (D) Scheme of the strand displacement reaction between the transcriptional switch and a unimolecular input strand. (E) Fluorescence signal obtained with the different variants in the absence and in the presence (30 nM) of the unimolecular input strand. (F) Ratio between the end-point fluorescence signals in the presence and absence of the input strand with the different variants. (G) Scheme of the co-localization-induced hybridization of the split input strands in the presence of an Ab-mimic DNA strand. (H) Fluorescence signal in the absence and presence (100 nM) of the Ab-mimic DNA strand at different split input strand concentrations. (I) Transcription kinetic traces in the absence (gray) and presence (black) of the Ab-mimic strand with a 30 nM concentration of split input strands. The experiments here were conducted at 25 °C in a 20 μL solution of a commercial transcription kit supplemented with the transcriptional switch module (100 nM), and the split input strands (30 nM) and the input strand (or Ab-mimic) were indicated. The transcription reaction was allowed to proceed for 120 min (or shorter time as indicated in panel I), and then, an aliquot was transferred to 100 μL of 10 mM Tris–HCl and 75 mM KCl, pH 7.4 solution containing 300 nM of TO-1, and the fluorescence signal measured after 15 min at 545 nm. The experimental values in this and in the following figures represent averages of at least three separate measurements, and the error bars reflect the standard deviations.

We initially focused on the thermodynamic optimization of the transcriptional switch module. Instrumental for the functioning of the sensing system is the need to have a switch that only induces transcription of the light-up RNA aptamer upon a strand displacement reaction with a functional input strand. We designed a set of transcriptional switches that share the same 17 nt T7-RNAP promoter sequence and the same hairpin structure with a 9 nt loop and a 6 nt stem (Figure 2A and Figure S1). The variants we designed have a variable length of the promoter sequence hidden in the stem-loop structure (from 1 to 17 nt) (Figure 2B and in Figure 2C, three variants are shown as an example). As the reporter light-up RNA aptamer produced by the transcriptional switch, we first employed the well-characterized Mango aptamer, a 39 nt RNA sequence that binds to a thiazole orange (TO-1) derivative resulting in an increase in its fluorescence signal.^24−26^ We tested the Mango aptamer signal in the absence and presence of a linear unimolecular DNA input strand that mimics the input strand that will be formed upon bivalent antibody binding to the antibody-responsive module (Figure 2D). This strand contains a toehold portion of 12 nt and an invading domain of 21 nt that allow efficient and rapid strand displacement reaction. We observed no signaling in the absence of the input strand with transcriptional switches in which the length of the accessible promoter domain is shorter than 12 nt. By further increasing the length of the accessible promoter domain, we observe higher signals that level off when this reaches 14 nt. In the presence of the input strand, instead, efficient transcription is observed for all switches with accessible promoter domains longer than 8 nt (Figure 2E,F). For shorter accessible promoter domains, no significant transcription is observed even after the input-induced conformational switch, likely because the presence of the nick in the formed promoter affects efficient T7-RNAP transcription.^27−30^ We found that a transcriptional switch with a 10 nt accessible promoter domain leads to the highest increase in Mango aptamer transcription in the presence of the input strand (Figure 2E,F).

With this optimized transcriptional switch, we then moved to the demonstration of the co-localization-induced transcription. To do this, we performed transcription experiments in the presence of the two strands composing the antibody-responsive module (i.e., split input strands). A 6 nt complementary portion between the two DNA strands was used (Figure 2G) as this length was previously demonstrated to be optimal to observe co-localization-induced hybridization.^31,32^ For this initial proof of principle, we used unmodified split input strands. As expected, under diluted conditions (<30 nM), the two strands do not lead to efficient transcription of the Mango aptamer, and only at saturating concentrations (>100 nM), we observe a signal comparable to that observed with a unimolecular input strand (Figure 2H). We then employed a bivalent DNA strand that acts as an antibody-mimic (Ab-mimic) and binds the tails of the two split input strands in a way similar to that expected from the binding of a bivalent antibody (Figure 2G). This Ab-mimic strand produces a light-up RNA aptamer with an efficiency similar to that of the complete unimolecular input strand even under diluted conditions (30 nM), thus supporting the proposed sensing scheme (Figure 2H,I and Figure S2).

### Programmable Transcriptional Switches for Antibody Detection

**Figure 3:**
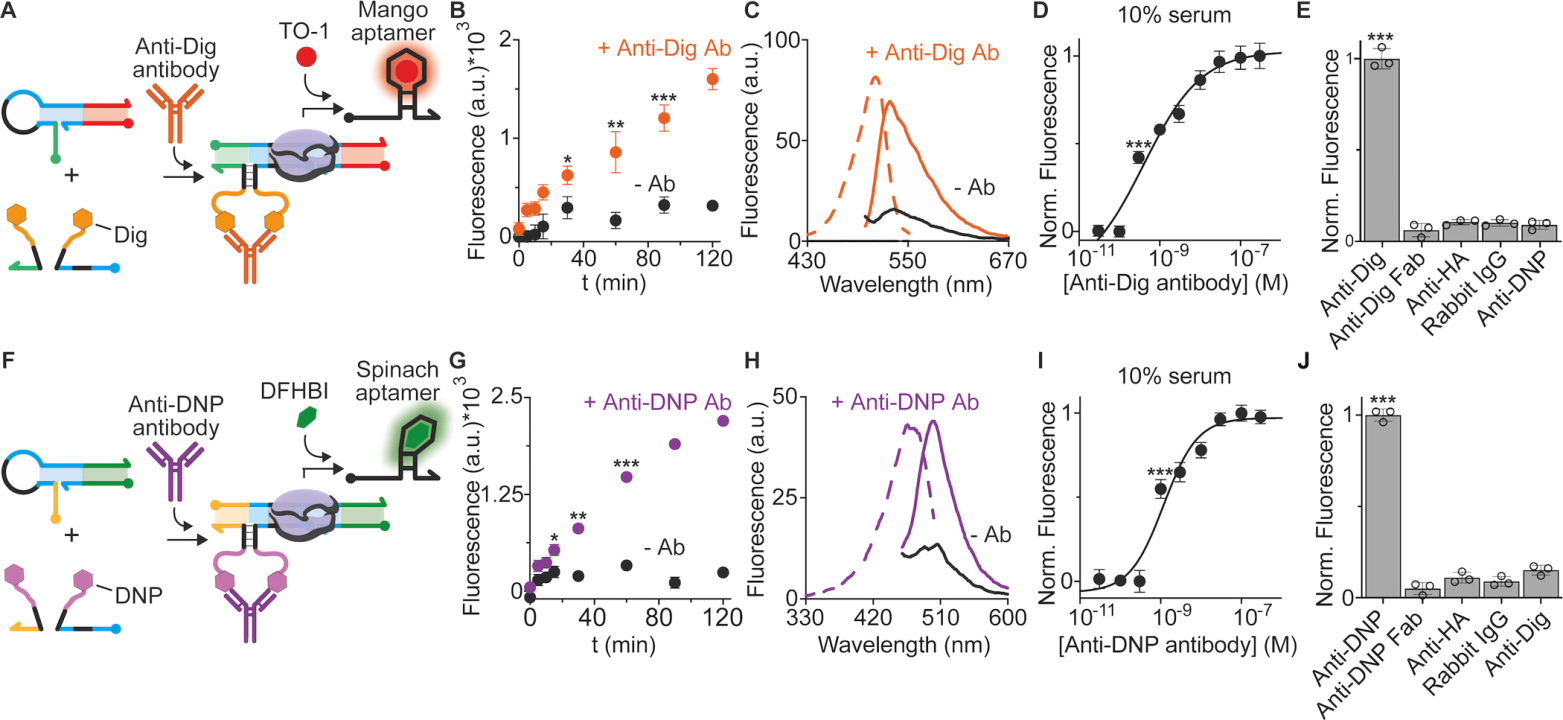
Antibody detection using programmable antibody-responsive transcriptional switches. (A) Scheme of the transcriptional switch for the detection of Anti-Dig antibodies. (B) Time course experiments showing the signal of the Mango aptamer-binding fluorophore (TO-1) obtained upon cell-free transcription experiments carried out in the absence and presence (100 nM) of the Anti-Dig antibody. (C) Emission and excitation spectra at the end-point (120 min) of reactions shown in panel (B). (D) Fluorescence signals in a 10% diluted bovine serum solution supplemented with increasing Anti-Dig antibody concentrations. (E) Fluorescence signal obtained with Anti-Dig antibodies and different non-specific targets (all at 100 nM) in 10% bovine serum. (F) Scheme of the transcriptional switch activated by the Anti-DNP antibody using DNP-conjugated DNA strands. (G) Time course experiments showing the signal of the Spinach aptamer-binding fluorophore (DFHBI) obtained upon cell-free transcription experiments carried out in the absence and presence (100 nM) of the Anti-DNP antibody. (H) Emission and excitation spectra at the end-point (120 min) of the reactions shown in panel (G). (I) Fluorescence signals in a 10% diluted bovine serum solution supplemented with increasing Anti-DNP antibody concentrations. (J) Fluorescence signal obtained with Anti-DNP antibodies and different non-specific targets (all at 100 nM) in 10% bovine serum. The experiments here were conducted at 25 °C in a 20 μL solution of a commercial transcription kit supplemented with the transcriptional switch module (100 nM), the antibody-responsive module (each at 30 nM), and the antibody (100 nM). For binding experiments (D, I), the antibody concentrations were varied from 0 to 300 nM, while the other components were kept at the same concentration as mentioned before. The transcription reaction was allowed to proceed for 120 min (or shorter time as indicated in panels B, G), and then, an aliquot was transferred to 100 μL of 10 mM Tris–HCl and 75 mM KCl, pH 7.4 solution containing the relevant dye, and the fluorescence signal measured after 15 min.

We then sought to demonstrate a proof of principle of transcriptional switches for antibody detection. To do this, we first employed as the recognition element (i.e., antigen) the small hapten digoxigenin (Dig) and we conjugated it at the two ends of the antibody-responsive module strands (Figure 3A). We performed transcription reactions with the transcriptional Mango switch module (100 nM) and the two Dig-conjugated DNA strands (at 30 nM) in the absence and presence of Anti-Dig monoclonal antibodies (Figure 3A). First, we observe that efficient Mango aptamer transcription is achieved only in the presence of Anti-Dig antibodies (100 nM) with output signals that start to level off after 120 min of reaction (Figure 3B). The fluorescence spectra obtained at the end of the transcription reaction in the presence of the Anti-Dig antibody show, as expected, the increase in the emission signal characteristic of the TO-1 dye when bound to the Mango aptamer (Figure 3C). The platform responds in a concentration-dependent fashion to Anti-Dig antibodies generating a 4-fold increase in fluorescence at the saturating concentration of Anti-Dig antibodies (100 nM, Figure S3) and achieving a detection limit in the low nanomolar range (*K*_D_ = 9.7 ± 0.4 nM, Figure S3) consistent with the reported bulk affinity of this antibody for its antigen.^33^ Of note, the transcriptional switch shows excellent sensitivities also in 10% bovine serum supplemented with increasing concentrations of Anti-Dig antibodies (Figure 3D). The platform is also highly specific, and no significant signal is observed in the presence of non-specific antibodies (at 100 nM) or in the presence of the Anti-Dig Fab fragment (that, being monovalent, cannot induce co-localization of the two antigen-conjugated strands) (Figure 3E).

One of the major advantages of our sensing strategy is that it is modular and versatile: changing the recognition elements allows us to potentially detect different target antibodies. To demonstrate this, we have employed responsive DNA strands conjugated with another recognition element (i.e., dinitrophenol, DNP) and designed a second transcriptional switch programmed to transcribe the Spinach aptamer as the reporter aptamer. With this new antibody-responsive transcriptional switch, we have measured Anti-DNP antibodies (IgG) (Figure 3F) reaching sensitivities and specificities similar to those observed for the Anti-Dig antibodies (Figure 3G–J and Figure S4).

Because the two antibody-responsive transcriptional switches we have characterized specifically to respond to a different antibody and induce the transcription of a specific light-up aptamer, we can use them in the same solution to achieve simultaneous detection of two antibodies. To do this, we mixed in the same solution the two transcriptional switches and added either one of the two antibodies or both of them (Figure S5). While the presence of a single antibody induces the fluorescence signal increase in the relevant dye-binding RNA aptamer, we observe high fluorescence signals for both dyes only in the presence of both antibodies (Figure S5).

To further demonstrate the versatility of our approach and the possible applications beyond sensing, we have employed a similar antibody-induced transcriptional switch for controlling the activity of a downstream target protein (Figure S6). To do so, we designed a new Anti-Dig transcriptional switch that, in the presence of the target antibody, transcribes an RNA aptamer that inhibits the activity of SP6 RNA polymerase.^34^ In the absence of the Anti-Dig antibody, the SP6-RNAP is fully active and induces transcription of the light-up Mango RNA aptamer from a second DNA template containing the SP6 promoter domain (Figure S6). In the presence of the specific Anti-Dig antibody, the transcribed aptamer inhibits the activity of the SP6-RNAP and we observe a strong reduction of the mango aptamer signal (Figure S6).

### Modular Transcriptional Switch for Antibody Detection

**Figure 4:**
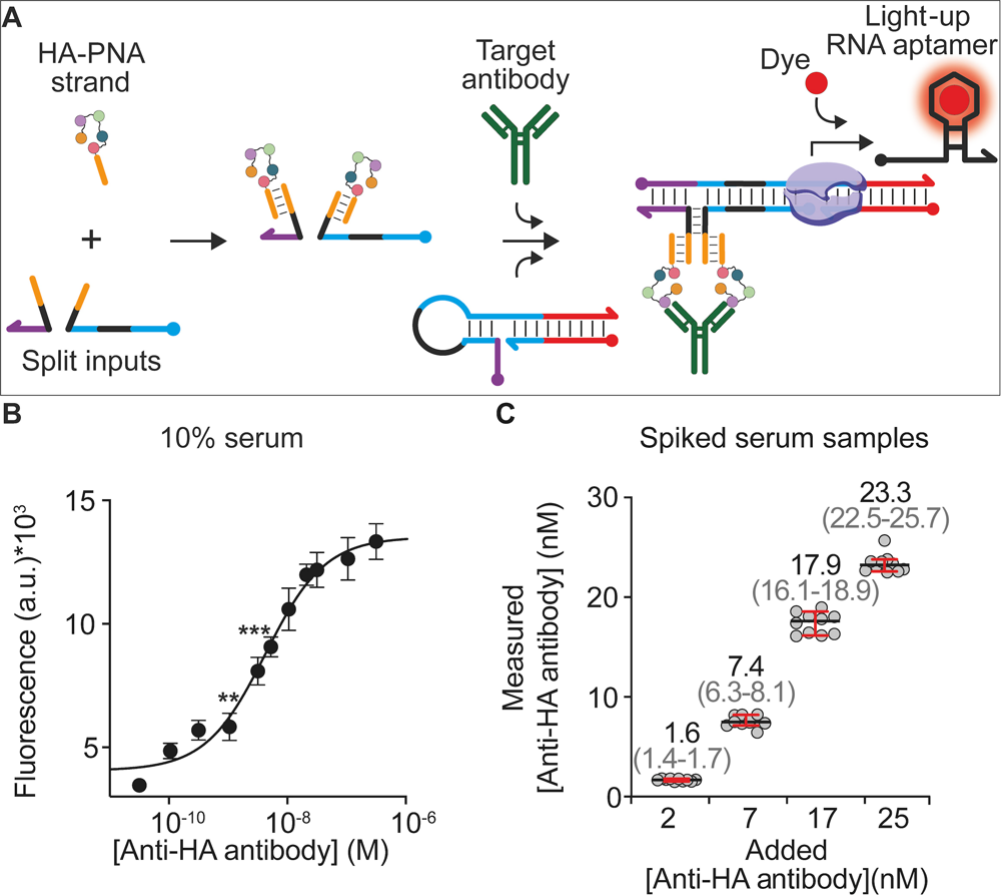
Modular transcriptional switch for Anti-HA antibody detection. (A) Modular version of the transcriptional switch to use peptide-PNA chimera strands as recognition elements of Anti-HA antibodies. (B) Fluorescence signal as a function of the Anti-HA concentration in a 10% diluted bovine serum solution. (C) Antibody quantification assessed by spiking blank serum samples with different known concentrations of the Anti-HA antibody (2, 7, 17, and 25 nM). Median values (black value and horizontal line) and 95% confidence intervals (gray values in parenthesis and red bars) are indicated. The experiments here were conducted at 25 °C in a 20 μL solution of a commercial transcription kit supplemented with the transcriptional switch module (100 nM), split input strands (each at 30 nM), HA-PNA chimera strands (100 nM), and different concentrations of the Anti-HA antibody (as indicated). The transcription reaction was allowed to proceed for 120 min, and then, an aliquot was transferred to 100 μL of 10 mM Tris–HCl and 75 mM KCl, pH 7.4 solution containing 300 nM of TO-1, and the fluorescence signal measured after 15 min.

After proof-of-principle demonstration of our transcriptional antibody-responsive switch, we sought to expand it to more clinically relevant antibodies. In this regard, it should be considered that antibodies produced upon infection are commonly raised against protein epitopes or peptides.^35,36^ From a synthetic point of view, the conjugation of peptides (and whole proteins) to DNA strands can be challenging and expensive. To overcome this limitation, we designed a modular version of our antibody-responsive transcriptional switch that can be easily adapted to the use of peptides as recognition elements. To do this, we reengineered the two antibody-responsive module strands to contain a 17 nt tail domain that can hybridize to a peptide-PNA strand (this presents a lower degree of difficulty of synthesis) (Figure 4A). With this modular approach, we have employed as the recognition element a nine-residue epitope enclosed in the human influenza hemagglutinin (HA protein)^37^ and recognized by the Anti-HA antibody (Figure 4A). This modular switch achieves sensitivities and specificities similar to its non-modular counterpart. More specifically, we found a sensitivity in the nanomolar range (*K*_D_ = 5 ± 1 nM in buffer, *K*_D_ = 4.1 ± 0.2 nM in 10% serum) (Figure 4B and Figures S7 and S8) and no signal change in the presence of non-specific antibodies (Anti-Dig, Anti-DNP, and Rabbit IgG) (Figure S9).

We have also evaluated the accuracy of the proposed method by determining the recovery percentages of spiked serum samples at four representative Anti-HA concentration levels (2, 7, 17, and 25 nM, *n* = 10 for each concentration) (Figure 4C). The results indicated good accuracy with recovery percentages between 77% (at 2 nM Anti-HA concentration, C.I. 95% = 1.4–1.7 nM) and 107% (at 7 nM Anti-HA concentration, C.I. 95% = 6.3–8.1 nM) (Figure 4C and Figure S10).

## Conclusions

In the present study, we report the development of cell-free transcriptional switches that can be activated by specific target antibodies. Our platform consists of DNA programmable modules that can respond to the presence of a target antibody and trigger the transcription of a light-up RNA aptamer. The modular nature of our platform makes it easily applicable to different antibodies with the expedient of changing the recognition element conjugated to the DNA-responsive module. By using this approach, we demonstrated efficient detection of three different antibodies with high sensitivity (low nanomolar levels) and excellent specificity (no significant cross-reactivity with non-specific antibodies). The transcriptional switch can also efficiently detect antibodies in complex biological matrices, such as blood serum. The approach we propose here is convenient as it requires only inexpensive synthetic antigen-conjugated DNA strands and commercially available transcription kits. We estimate a cost of $0.5–1 per test^39^ in the current detection scheme. Furthermore, while the approach relies on fluorescence detection (which can be impractical for point-of-care use), it can be easily adapted to electrochemical detection with the expedient of using DNA-based disposable sensors.^40,41^

This work builds on previous advancements in the field of cell-free nucleic acid diagnostics^5−14^ and demonstrates that *in vitro* transcription/translation processes combined with programmable responsive nucleic acid devices can be conveniently applied for the detection of a wide range of clinical markers. Considering the importance of the detection of antibodies in controlling infectious diseases and their growing therapeutic use,^42−44^ this and similar approaches can lead to transformative improvements in antibody detection technologies applied to clinical problems. Despite the above advantages, the approach is not without limitations and remains intrinsically more complicated than sensing approaches that rely on direct signal translation of target binding (either electrochemical or optical). For example, the response time of the system (ca. 2 h) is quite long and it is mostly determined by the transcription reaction time and by the kinetics of dye-aptamer binding. Transcription time could be reduced to 15–30 min without a significant loss of sensitivity (see Figure 3B,G), and the overall analysis procedure could be simplified with the use of real-time measurement of the RNA transcript as reported elsewhere.^38^

The antibody-controlled transcriptional switches reported here can also have implications beyond sensing and diagnostics. We have demonstrated this by programming the antibody-induced transcription of an RNA aptamer that inhibits a second downstream protein. Similar systems can be applied for the targeted transcription of therapeutic or functional RNA sequences in the presence of specific target antibodies opening to a wide range of possible clinical applications.

## Experimental Section

### Reagents and Materials

Reagent-grade chemicals (NaCl, KCl, MgCl_2_, Trizma base, Trizma hydrochloride, and DEPC-treated water) were purchased from Sigma-Aldrich (St Louis, Missouri) and used without further purifications. TO-1-3PEG-Biotin (TO-1) fluorophore was purchased from abmgood (Canada, cat #G955), and DFBHI-1 T was purchased from TOCRIS Biosciences (Bristol, UK) cat. no. 1625/10. Sheep polyclonal Anti-Dig antibodies were purchased from Roche Diagnostic Corporation, Germany (cat #11333089001), mouse monoclonal Anti-DNP antibodies and rabbit polyclonal Anti-mouse IgG antibodies were purchased from Sigma-Aldrich, USA, (cat #D9656 and #06-371), and Anti-Dig Fab and Anti-HA antibodies were purchased from Roche Diagnostic Corporation, (Germany) (cat #11214667001). All the antibodies were aliquoted and stored at 4 °C for immediate use or at −20 °C for long-term storage.

### Oligonucleotides

HPLC-purified oligonucleotides were purchased from Metabion (Planegg, Germany) or Biosearch Technologies (Risskov, Denmark). The split input oligonucleotide strands that activate the transcriptional switches were modified with digoxigenin or dinitrophenyl moieties. PNA sequences modified with the HA peptide were obtained from Panagene (South Korea). All the sequences of the transcriptional switch variants and antibody-responsive modules are reported in the Supporting Information document.

### Cell-Free Transcription Reactions

All transcription reactions were performed using a ThermoFisher TranscriptAid T7 high yield transcription kit (ref. K0441) following the recommended manufacturer protocols. More specifically, we have prepared the 20 μL solution of the commercial transcription kit so that they contain the transcriptional switch module (100 nM), the antibody-responsive module (30 nM), and the antibody (when indicated). The transcription reaction was allowed to proceed for 120 min after polymerase addition. An aliquot of the transcription reaction solution (1 or 5 μL) was then transferred to 100 μL of 10 mM Tris–HCl and 75 mM KCl, pH 7.4 solution containing the relevant dye (either TO-1, 300 nM or DFHBI, 1 μM), and the fluorescence signal measured after 15 min.

### Fluorescence Experiments

Fluorescence measurements were obtained in a Tecan M200pro plate reader using top reading mode with black, flat bottom non-binding 96-well plates. For the Mango RNA aptamer (TO-1 dye), the excitation wavelength was fixed at 506 (±9) nm and the emission wavelength at 545 (±20) nm. For the Spinach RNA aptamer (DFHBI dye), the excitation wavelength was fixed at 455 (±9) nm and the emission wavelength at 506 (±20) nm. Curve fitting at different concentrations of the target antibody was obtained using Prism-GraphPad software and its built-in Hill function with the following Lavenberg–Marquardt iteration algorithm:
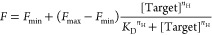
where *F*_min_ and *F*_max_ are the minimum and maximum fluorescence values, respectively, *K*_D_ is the equilibrium antibody concentration at the half-maximum signal, *n*_H_ is the Hill coefficient, and [Target] is the concentration of the specific antibody added.

To allow for a more ready interpretation of the results, fluorescence signals obtained have been normalized on a 0–1 scale using the following formula:
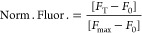
where *F*_T_ is the fluorescence signal obtained in the presence of the target antibody, *F*_0_ is the fluorescence signal obtained in the absence of the target, and *F*_max_ represents the maximum fluorescence signal of the platform at a saturating concentration of the target (Rel. Fluor. = 1). The experimental values represent averages of three separate measurements, and the error bars reflect the standard deviations.

Statistical analysis was performed with Prism GraphPad 9vs using two-tailed unpaired Student’s *t* test, and the *p*-value ranges are indicated with black asterisks (*** < 0.001, ** = 0.001–0.01, * = 0.01–0.05).

### Anti-HA Antibody Quantification

Recovery experiments were performed by adding known amounts of the Anti-HA antibody into blank serum samples. Anti-HA antibody concentration values were obtained by replacing the observed fluorescence values in the equation of the binding curve reported in Figure 4B linear in the equation of the binding curve reported in Figure 4B. Differences between spiked serum concentrations were calculated with a two-way ANOVA test.

## Supporting Information Available

Additional experimental details and DNA sequences used; predicted secondary structures for the different variants; binding curves for Anti-Dig, Anti-DNP, and Anti-HA antibodies in buffer solution; and simultaneous Anti-Dig and Anti-DNP antibody detection (PDF)
