# Supplementary material for: Programmable Cell-Free Transcriptional Switches for Antibody Detection
Source: J Am Chem Soc. 2022 Mar 22;144(13):5820–6. doi: 10.1021/jacs.1c11706 (PMC8990998; doi:10.1021/jacs.1c11706)
Supplement: Supplementary file 1 — ja1c11706_si_001.pdf [file ja1c11706_si_001.pdf]

Supplementary information:

Programmable cell-free transcriptional switches for antibodies detection

Aitor Patino Diaz,[1,#] Sara Bracaglia,[1,#] Simona Ranallo,[1] Tania Patino,[1] Alessandro Porchetta,[1] and Francesco Ricci[1,*]

[1]Chemistry Department, University of Rome, Tor Vergata, Via della Ricerca Scientifica, 00133, Rome, Italy.

Supporting Information:

Supplementary Methods

DNA Sequences

All sequences were designed using Nupack or IDT oligoanalyzer tools. [1,2]

1) Anti-Dig transcriptional switches

Name	Sequence
Sense strand #1	5'- **AGA ATG GGA TAG** *TAT AGT* TAA TAC GAC TC *ACT ATA* GGG CCG CCG GTA CCT CCG AAG GGA CGG TGC GGA GAG GAG AGG GGG CAC TGG GC -3'
Sense strand #2	5'- **AGA ATG GGA TAG** *TAG TGA* TAA TAC GAC *TCA CTA* TA GGG CCG CCG GTA CCT CCG AAG GGA CGG TGC GGA GAG GAG AGG GGG CAC TGG GC -3'
Sense strand #3	5'- **AGA ATG GGA TAG** *GTG AGT* AA TAA TAC G *ACT CAC* TAT A GGG CCG CCG GTA CCT CCG AAG GGA CGG TGC GGA GAG GAG AGG GGG CAC TGG GC -3'
Sense strand #4	5'- **AGA ATG GGA TAG** *GAG TCG* AAA A TAA TA *CGA CTC* ACT ATA GGG CCG CCG GTA CCT CCG AAG GGA CGG TGC GGA GAG GAG AGG GGG CAC TGG GC -3'
Sense strand #5	5'- **AGA ATG GGA TAG** *GTC GTA* ATA TAC TAA *TAC GA*C TCA CTA TA GGG CCG CCG GTA CCT CCG AAG GGA CGG TGC GGA GAG GAG AGG GGG CAC TGG GC -3'
Sense strand #6	5'- **AGA ATG GGA TAG** *CGT ATT* TCA TAT AC T *AAT ACG* ACT CAC TAT A GGG CCG CCG GTA CCT CCG AAG GGA CGG TGC GGA GAG GAG AGG GGG CAC TGG GC -3'
Sense strand #7	5'- **AGA ATG GGA TAG** *TAT TAC* TCA TAT ACT *G TAA TA* CGA CTC ACT ATA GGG CCG CCG GTA CCT CCG AAG GGA CGG TGC GGA GAG GAG AGG GGG CAC TGG GC -3'
Sense strand #8	5'- **AGA ATG GGA TAG** *TTA GTA* AAA TAA AAA *TAC TAA* TAC GAC TCA CTA TA GGG CCG CCG GTA CCT CCG AAG GGA CGG TGC GGA GAG GAG AGG GGG CAC TGG GC -3'

Sense strand #9	5'- **AGA ATG GGA TAG** *AGT ATA* AAA TAA AAA *TAT AC T* AA TAC GAC TCA CTA TA GGG CCG CCG GTA CCT CCG AAG GGA CGG TGC GGA GAG GAG AGG GGG CAC TGG GC -3'
Antisense strand #1	5'- GCC CAG TGC CCC CTC TCC TCT CCG CAC CGT CCC TTC GGA GGT ACC GGC GGC CC -3'
Antisense strand #2	5'- GCC CAG TGC CCC CTC TCC TCT CCG CAC CGT CCC TTC GGA GGT ACC GGC GGC CC TA -3'
Antisense strand #3	5'- GCC CAG TGC CCC CTC TCC TCT CCG CAC CGT CCC TTC GGA GGT ACC GGC GGC CC TAT A -3'
Antisense strand #4	5'- GCC CAG TGC CCC CTC TCC TCT CCG CAC CGT CCC TTC GGA GGT ACC GGC GGC CC TAT AGT -3'
Antisense strand #5	5'- GCC CAG TGC CCC CTC TCC TCT CCG CAC CGT CCC TTC GGA GGT ACC GGC GGC CC TAT AGT GA -3'
Antisense strand #6	5'- GCC CAG TGC CCC CTC TCC TCT CCG CAC CGT CCC TTC GGA GGT ACC GGC GGC CC TAT AGT GAG T -3'
Antisense strand #7	5'- GCC CAG TGC CCC CTC TCC TCT CCG CAC CGT CCC TTC GGA GGT ACC GGC GGC CC TAT AGT GAG TCG -3'
Antisense strand #8	5'-GCC CAG TGC CCC CTC TCC TCT CCG CAC CGT CCC TTC GGA GGT ACC GGC GGC CC TAT AGT GAG TCG TA-3'
Antisense strand #9	5'-GCC CAG TGC CCC CTC TCC TCT CCG CAC CGT CCC TTC GGA GGT ACC GGC GGC CC TAT AGT GAG TCG TAT T-3'
Linear input #1	5'-<u>TAT AGT GAG TCG TAT TAA CTA TA</u> **CTA TCC CAT TCT** -3'
Linear input #2	5'-<u>TAG TGA GTC GTA TTA TCA CTA</u> **CTA TCC CAT TCT**-3'
Linear input #3	5'-<u>GTG AGT CGT ATT ATT ACT CAC</u> **CTA TCC CAT TCT**-3'
Linear input #4	5'-<u>GAG TCG TAT TAT TTT CGA CTC</u> **CTA TCC CAT TCT**-3'
Linear input #5	5'-<u>GTC GTA TTA GTA TAT TAC GAC</u> **CTA TCC CAT TCT**-3'
Linear input #6	5'- <u>CGT ATT AGT ATA TGA AAT ACG</u> **CTA TCC CAT TCT** -3'
Linear input #7	5'- <u>TAT TAC AGT ATA TGA GTA ATA</u> **CTA TCC CAT TCT** -3'
Linear input #8	5'- <u>TTA GTA TGT ATA TGA TAC TAA</u> **CTA TCC CAT TCT** -3'
Linear input #9	5'- <u>AGT ATA TTT TTA TTT TAT ACT</u> **CTA TCC CAT TCT** -3'
Split input #1	5'- GTC ACC GCA AAA TAA GAA TTT TTT TTT T *CAG ACG* **CTA**

	**TCC CAT TCT** -3'
Split input #2	5'- CGT ATT AGT ATA TGA AAT ACG *CGT CTG* TTT TTT TTT TAA GAA TAA AAC GCC ACT G -3'
Antigen-conjugated strand #1	(DIG) 5'- TTT TTT TTT TTT TTT TTT TT *CAG ACG* **CTA TCC CAT TCT** -3'
Antigen-conjugated strand #2	5'- CGT ATT AGT ATA TGA AAT ACG *CGT CTG* TTT TTT TTT TTT TTT TTT TT -3' (DIG)
Ab-mimic	5'- CAG TGG CGT TTT ATT CTT TTT TTT TTT TTT TTT CTT ATT TTG CGG TGA C -3'

Here the *italic* bases denote the stem forming portion, the underlined sequences represent the invading portion, while the **bold** bases denotes the toehold domain. In the antigen-conjugated splits digoxigenin (Dig) was introduced via EDC/NHS coupling to an amine attached via a 5-carbon linker on the 5' end or on the 3' end.

2) Anti-DNP transcriptional switch

**Name**	**Sequence**
Sense strand	5'- **TAG AAT GAG GGA** *CGT ATT* TCC TAA TAT *AAT ACG* ACT CAC TAT AGG GTG AAG GAC GGG TCC GTT CGC GTT GAG TAG AGT GTG AGC TCC -3'
Antisense strand	5'- GGA GCT CAC ACT CTA CTC AAC GCG AAC GGA CCC GTC CTT CAC CC TAT AGT GAG T -3'
Linear input	5'- CGT ATT ATA TTA GGA AAT ACG **TCC CTC ATT CTA** -3'
Split input#1	(DNP) 5'- TTT TTT TTT TTT TTT TTT TT *CAG ACG* **TCC CTC ATT CTA** -3'
Split input #2	5'- CGT ATT ATA TTA GGA AAT ACG *CGT CTG* TTT TTT TTT TTT TTT TTT TT -3' (DNP)

Here the *italic* bases denote the stem forming portion, the underlined sequences represent the invading portion, while the **bold** bases denotes the toehold domain. In the antigen-conjugated splits Dinitrophenol (DNP) was attached via a triethylene glycol (TEG) spacer arm on either the 5' or the 3' terminus of the appropriate oligonucleotide.

3) Anti-HA transcriptional switch

Name	Sequence
Split input#1	3'- GTC ACC GCA AAA TAA GA TTT -5' 5'- *CAG ACG* **CTA TCC CAT TCT** -3'
Split input #2	5'- <u>CGT ATT AGT ATA TGA AAT ACG</u> *CGT CTG* TTT AGA ATA AAA CGC CAC TG -3'
Peptide-PNA strand	N_{term}-(YPYDVPDYA)- CAG TGG CGT TTT ATT CT- C_{term}

Here the *italic* bases denote the stem forming portion, the <u>underlined</u> sequences represent the invading portion, while the **bold** bases denotes the toehold domain. The sequence in parentheses represents the selected peptide epitope portions that is terminally conjugated to PNA.

4) Templates and strands for transcriptional switch inhibiting SP6 polymerase

Name	Sequence
Sense strand-T7:Sp6inhibitory aptamer	5'- **AGA ATG GGA TAG** *CGT ATT* TCA TAT AC T *AAT ACG* ACT CAC TAT A <u>GGG AGA GTT GCT TGG AAT GCG TTA TAG TCT CTT AGG TGT GTT CGC ACA CCA CTC TC</u> -3'
Antisense strand-T7:Sp6inhibitory aptamer	5'- <u>GGA GAG TGG TGT GCG AAC ACA CCT AAG AGA CTA TAA CGC ATT CCA AGC AAC TCT CCC</u> TAT AGT GAG T -3'
Sense strand-Sp6:Mango aptamer	5'- TGA GGA ATA CAT ATA C ATT TAG GTG ACA CTA TAG <u>GGG CCG CCG GTA CCT CCG AAG GGA CGG TGC GGA GAG GAG AGG GGG CAC TGG GC</u> -3'
Antisense strand-Sp6:Mango aptamer	5' - <u>GCC CAG TGC CCC CTC TCC TCT CCG CAC CGT CCC TTC GGA GGT ACC GGC GGC CCC</u> TAT AGT GTC ACC TAA AT G TAT ATG TAT TCC TCA - 3'

Here the *italic* bases denote the stem forming portion, the **bold** bases denotes the toehold domain. The underlined sequences represent the gene transcribing for the corresponding aptamer.

Supplementary Figures

Legend: ●A ●C ●G ●T

Variant	Free energy (kcal/mol)	Secondary structure
#1	-105	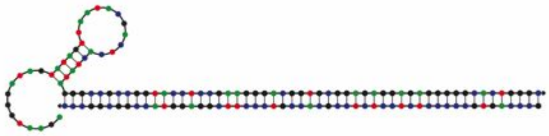
#2	-107	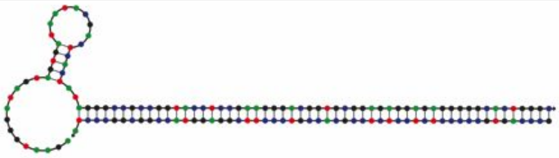
#3	-111	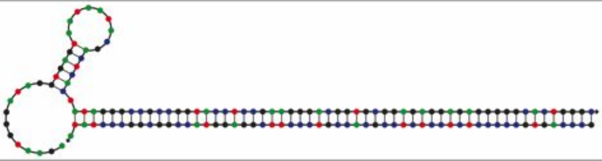
#4	-115	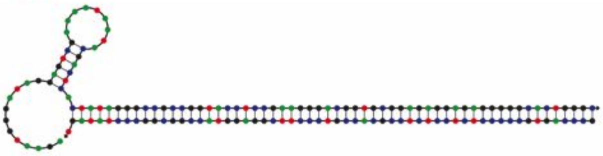
#5	-117	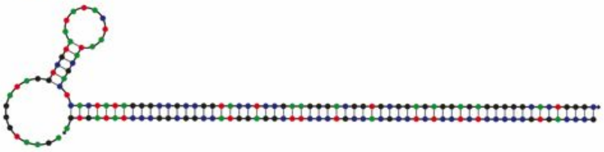
#6	-119	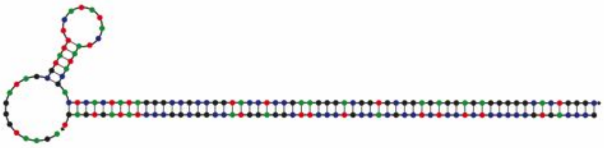
#7	-123	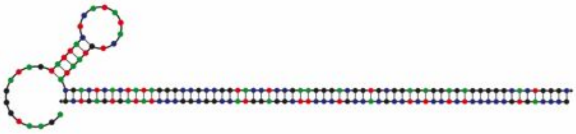
#8	-124	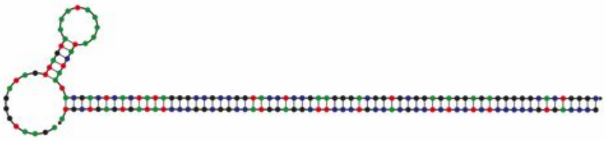
#9	-127	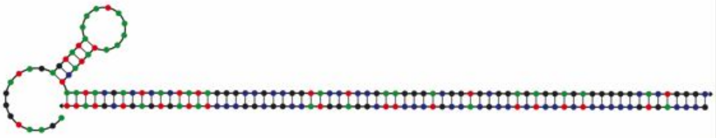

Figure S1. Predicted secondary structures and associated free energies provided by NUPACK for the different transcriptional switch variants. The switches share the same 17-nt T7 RNAP promoter sequence and the same hairpin structure with a 9-nt loop and a 6-nt

stem. The variants have a variable length of the promoter sequence hidden in the stem-loop structure (from 1 to 17 nt). The length of the single-stranded toehold domain is kept always the same (12-nt).

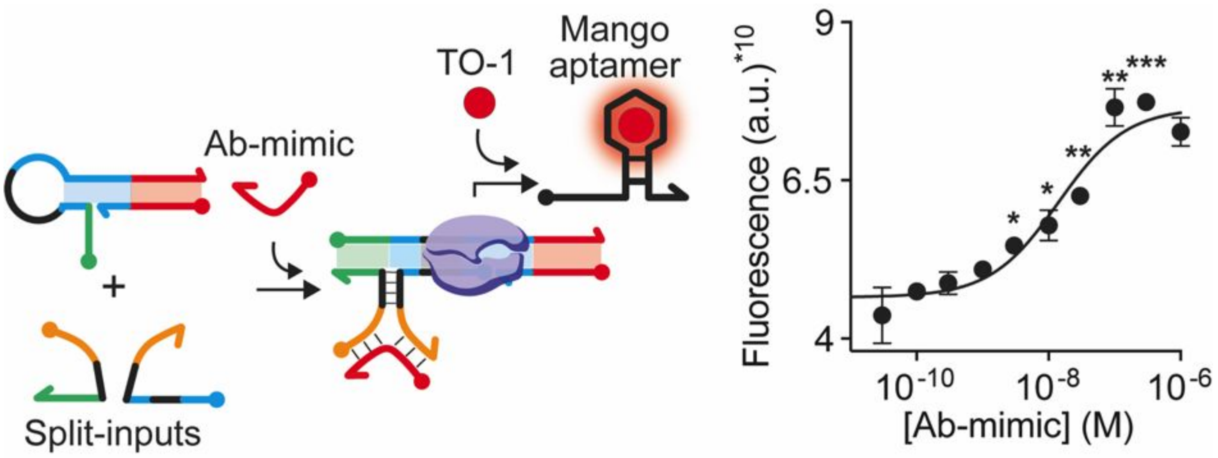

Figure S2. Fluorescence signals of the Mango aptamer at increasing concentration of Ab-mimic DNA strand. The experiment was conducted at 25°C in a 20 µL solution of a commercial transcription kit supplemented with the transcriptional switch module (100 nM), split input strands (30 nM). The transcription reaction was allowed to proceed for 120 min and then an aliquot was transferred to 100 µL of 10 mM Tris-HCl and 75 mM KCl, pH 7.4 solution containing 300 nM of TO-1 and the fluorescence signal measured after 15 min at 545 nm.

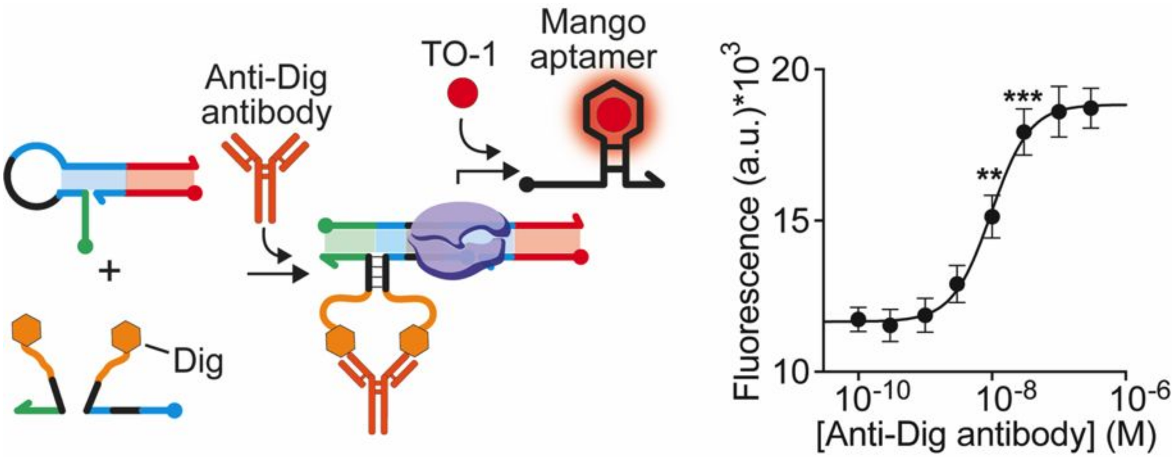

Figure S3. Anti-Dig antibody detection by using the antibody-responsive transcriptional switch that comprise digoxigenin as antigen and transcribes the Mango aptamer. The experiment was conducted at 25°C in a 20 µL solution of a commercial transcription kit supplemented with the transcriptional switch module (100 nM), antibody-responsive module (30 nM). The transcription reaction was allowed to proceed for 120 min and then an aliquot was transferred to 100 µL of 10 mM Tris-HCl and 75 mM KCl, pH 7.4 solution containing 300 nM of TO-1 and the fluorescence signal measured after 15 min at 545 nm.

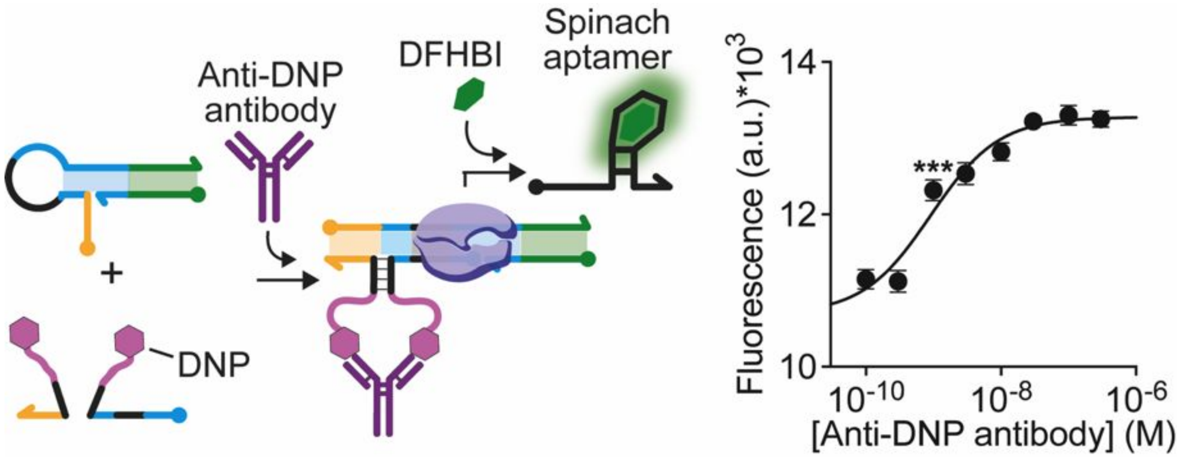

Figure S4. Anti-DNP antibody detection by using the antibody-responsive transcriptional switch that comprise dinitrophenol as antigen and transcribes the Spinach aptamer. The experiment was conducted at 25°C in a 20 μL solution of a commercial transcription kit supplemented with the transcriptional switch module (100 nM), the antibody-responsive module (30 nM). The transcription reaction was allowed to proceed for 120 min and then an aliquot was transferred to 100 μL of 10 mM Tris-HCl and 75 mM KCl, pH 7.4 solution containing 1 μM of DFHBI and the fluorescence signal measured after 15 min at 506 nm.

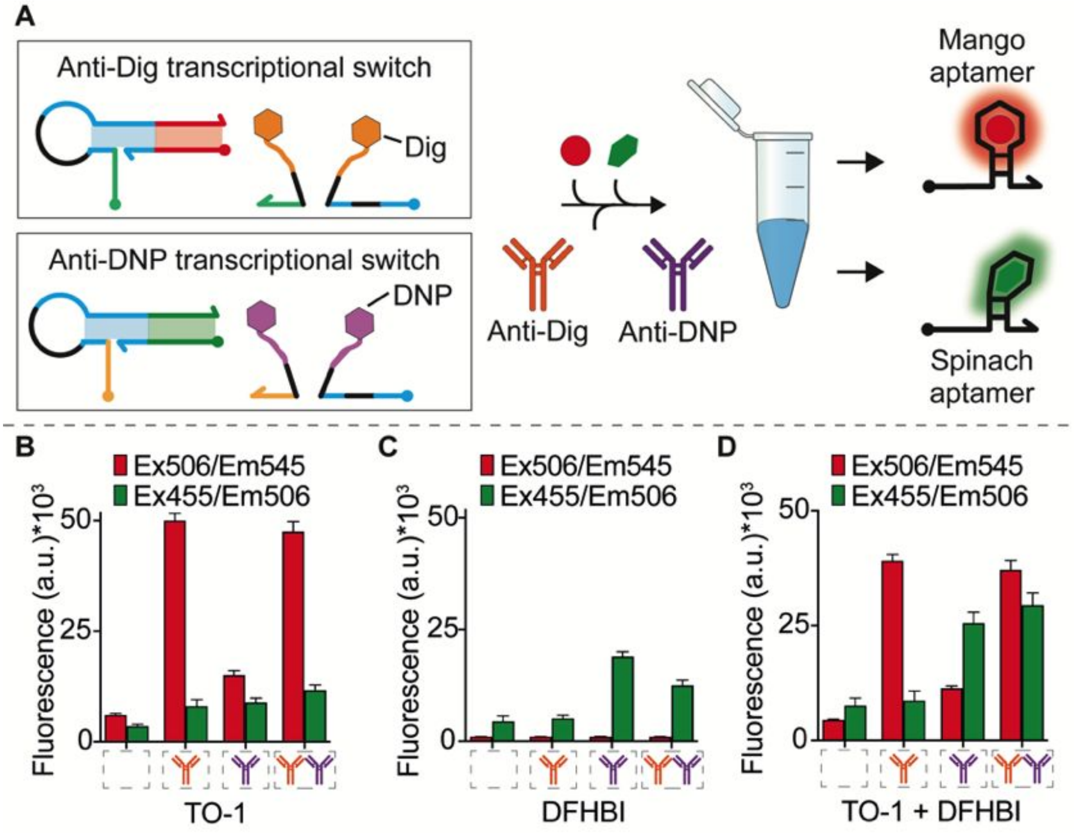

Figure S5. Simultaneous Anti-Dig and Anti-DNP antibodies detection. (A) Two antibody-responsive transcriptional switches specific for Anti-Dig and Anti-DNP and inducing the transcription of a Mango RNA aptamer or a Spinach RNA aptamer, respectively, were used in the same reaction solution. The fluorescence signal was measured at 506/545 nm and 455/506 nm in the presence of TO-1 (B), DFHBI (C) and both dyes (D) in the presence of various combinations of Anti-Dig and Anti-DNP antibodies. The experiments were conducted at 25°C in a 20 µL solution of a commercial transcription kit supplemented with the two transcriptional switch modules (each at 100 nM), antibody-responsive modules (each at 30 nM) and saturating concentrations of antibodies (100 nM). The transcription reaction was allowed to proceed for 120 min and then an aliquot was transferred to 100 µL of 10 mM Tris-HCl and 75 mM KCl, pH 7.4 solution containing 300 nM of TO-1 and 1 µM of DFHBI. The fluorescence signal was measured after 15 min.

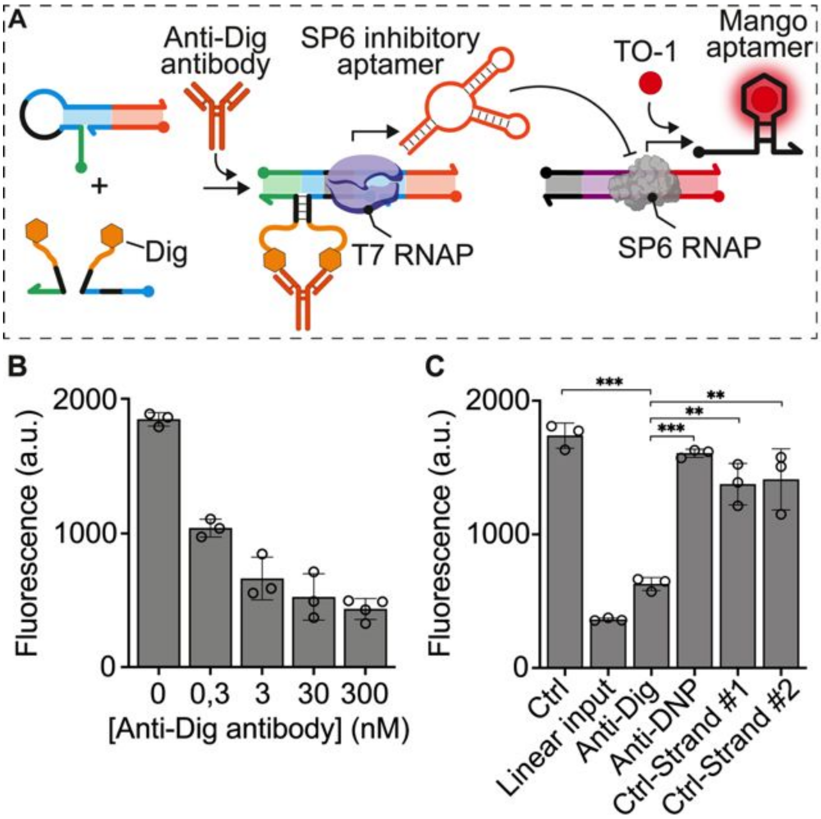

Figure S6. Antibody-induced transcription of an RNA aptamer that inhibits SP6-RNA-polymerase (SP6-RNAP) (A) cartoon showing the two DNA templates used: a first antibody-responsive transcriptional switch (top, left) specific for Anti-Dig antibody where a T7 promoter (blue) transcribes a SP6 inhibitory aptamer (orange), and second template (right) where a SP6 promoter (violet) transcribes a Mango RNA aptamer (red). When the target Anti-Dig antibody is present the transcribed RNA aptamer inhibits SP6 RNAP and the transcription of the Mango aptamer is reduced. (B) Fluorescence signals obtained by varying Anti-Dig antibody concentrations. (C) Fluorescence signals observed in the presence of Anti-Dig antibodies (100 nM), non-specific Anti-DNP antibodies and with different control experiments. The experiments were conducted at 25°C in a 20 µL solution of a commercial T7 RNAP transcription kit supplemented with SP6 RNAP (2 U/µL), the two DNA templates (each at 100 nM), antibody-responsive modules (each at 30 nM) and different concentrations of Anti-Dig antibody (as indicated). For specificity study (panel C) the antibodies concentrations were kept at 3 nM. The transcription reaction was allowed to proceed for 120 min and then an aliquot was transferred to 100 µL of 10 mM Tris-HCl and 75 mM KCl, pH 7.4 solution containing 300 nM of TO-1. The fluorescence signal was measured after 15 min.

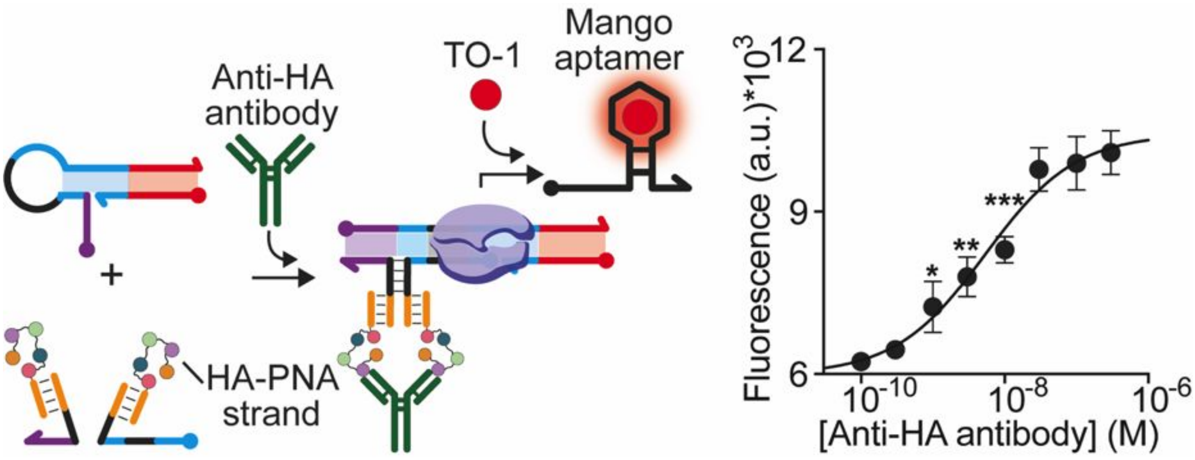

Figure S7. Modular transcriptional switch for the detection of Anti-HA antibody. The experiment was conducted at 25°C in a 20 µL solution of a commercial transcription kit supplemented with the transcriptional switch module (100 nM), split input strands (30 nM), HA-PNA chimera strands (100 nM). The transcription reaction was allowed to proceed for 120 min and then an aliquot was transferred to 100 µL of 10 mM Tris-HCl and 75 mM KCl, pH 7.4 solution containing 300 nM of TO-1 and the fluorescence signal measured after 15 min at 545 nm.

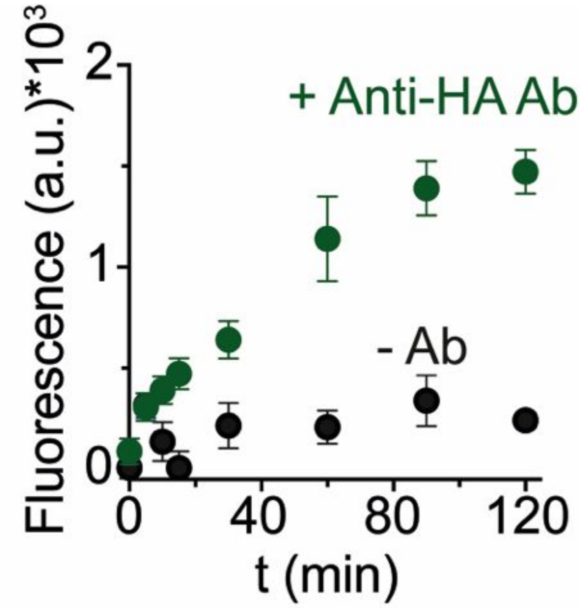

Figure S8. Time course experiments showing the signal of the Mango aptamer-binding fluorophore (TO-1) obtained upon cell-free transcription experiments carried out in the absence and presence (100 nM) of Anti-HA antibody. The experiment was conducted at 25°C in a 20 µL solution of a commercial transcription kit supplemented with the transcriptional switch module (100 nM), split input strands (30 nM), HA-PNA chimera strands (100 nM). The transcription reaction was allowed to proceed for 120 min and then an aliquot was transferred to 100 µL of 10 mM Tris-HCl and 75 mM KCl, pH 7.4 solution containing 300 nM of TO-1 and the fluorescence signal measured after 15 min at 545 nm.

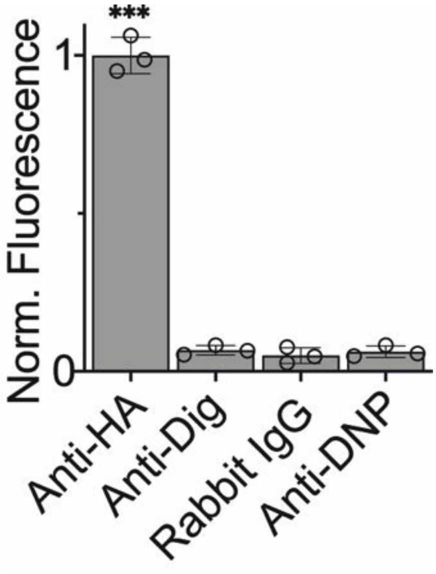

Figure S9. Normalized fluorescence signal observed with Anti-HA antibody and different non-specific antibodies (all at 100 nM). The experiment was conducted at 25°C in a 20 µL solution of a commercial transcription kit supplemented with the transcriptional switch module (100 nM), split input strands (30 nM), HA-PNA chimera strands (100 nM). The transcription reaction was allowed to proceed for 120 min in 10% serum and then an aliquot was transferred to 100 µL of 10 mM Tris-HCl and 75 mM KCl, pH 7.4 solution containing 300 nM of TO-1 and the fluorescence signal measured after 15 min at 545 nm.

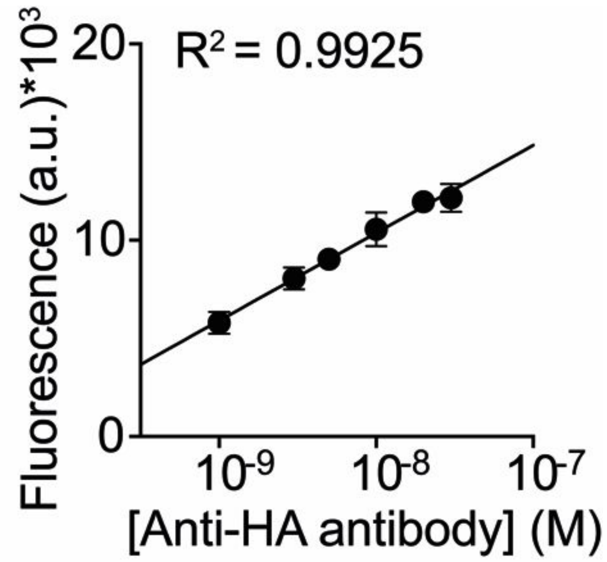

Figure S10. Linear dynamic range of Anti-HA antibody detection is between 1 and 30 nM (R^2 = 0.9925, n = 3). The experiment was conducted at 25°C in a 20 µL solution of a commercial transcription kit supplemented with the transcriptional switch module (100 nM), split input strands (30 nM), HA-PNA chimera strands (100 nM). The transcription reaction was allowed to proceed for 120 min in 10% serum and then an aliquot was transferred to 100 µL of 10 mM Tris-HCl and 75 mM KCl, pH 7.4 solution containing 300 nM of TO-1 and the fluorescence signal measured after 15 min at 545 nm.

Supplementary References

(1) Zadeh, J. N.; Steenberg, C. D.; Bois, J. S.; Wolfe, B. R.; Pierce, M. B.; Khan, A. R.; Dirks, R. M.; Pierce, N. A. NUPACK: Analysis and Design of Nucleic Acid Systems. *J. Comput. Chem.* **2011**, *32* (1), 170–173.

(2) Owczarzy, R.; Tataurov, A. V.; Wu, Y.; Manthey, J. A.; McQuisten, K. A.; Almabrazi, H. G.; Pedersen, K. F.; Lin, Y.; Garretson, J.; McEntaggart, N. O.; Sailor, C. A.; Dawson, R. B.; Peek, A. S. IDT SciTools: A Suite for Analysis and Design of Nucleic Acid Oligomers. *Nucleic. Acids. Res.* **2008**, *36* (Web Server issue), W163-169.
